# The Effectiveness of Digital Apps Providing Personalized Exercise Videos: Systematic Review With Meta-Analysis

**DOI:** 10.2196/45207

**Published:** 2023-07-13

**Authors:** Thomas Davergne, Philippe Meidinger, Agnès Dechartres, Laure Gossec

**Affiliations:** 1 Physical Medicine and Rehabilitation Department Assistance Publique – Hôpitaux de Paris Lariboisière-Fernand-Widal Université Paris Cité, Institut national de la santé et de la recherche médicale, Biologie de l'os et du cartilage Paris France; 2 Université Grenoble Alpes Centre national de la recherche scientifique, VetAgro Sup, Grenoble Institut polytechnique de Grenoble Grenoble France; 3 Sorbonne Université, INSERM, Institut Pierre Louis d'Epidémiologie et de Santé Publique, AP-HP, Hôpital Pitié-Salpêtrière Département de Santé Publique, 75013 Paris France; 4 Rheumatology Department Pitié-Salpêtrière Hospital, Assistance Publique – Hôpitaux de Paris, Institut Pierre Louis d'Epidémiologie et de Santé Publique, Institut national de la santé et de la recherche médicale Sorbonne Université Paris France

**Keywords:** app, exercise program, telerehabilitation, rehabilitation, disability, disabilities, digital care, web-based, exercise, physical activity, fitness, health app, HRQoL, QoL, quality of life, physical therapy, physiotherapy, systematic review, review method, adherence, meta-analysis, meta-analyses

## Abstract

**Background:**

Among available digital apps, those providing personalized video exercises may be helpful for individuals undergoing functional rehabilitation.

**Objective:**

We aimed to assess the effectiveness of apps providing personalized video exercises to support rehabilitation for people with short- and long-term disabling conditions, on functional capacity, confidence in exercise performance, health care consumption, health-related quality of life, adherence, and adverse events.

**Methods:**

In this systematic review, we searched MEDLINE, CENTRAL, and Embase databases up to March 2022. All randomized controlled trials evaluating the effect of apps providing personalized video exercises to support rehabilitation for any condition requiring physical rehabilitation were included. Selection, extraction, and risk of bias assessment were performed by 2 independent reviewers. The primary outcome was functional capacity at the end of the intervention. The secondary outcomes included confidence in exercise performance, care consumption, health-related quality of life, adherence, and adverse events. A meta-analysis was performed where possible; the magnitude of the effect was assessed with the standardized mean difference (SMD).

**Results:**

From 1641 identified references, 10 papers (n=1050 participants, 93% adults) were included: 7 papers (n=906 participants) concerned musculoskeletal disorders and 3 (n=144 participants) concerned neurological disorders. Two (n=332 participants) were employee based. The apps were mostly commercial (7/10); the videos were mostly elaborated on by a physiotherapist (8/10). The duration of app use was 3-48 weeks. All included studies had a high overall risk of bias. Low-quality evidence suggested that the use of apps providing personalized video exercises led to a significant small to moderate improvement in physical function (SMD 0.35, 95% CI 0.19-0.51; *P*het=.86; *I*^2^=0%) and confidence in exercise performance (SMD 0.67; 95% CI 0.37-0.96; *P*het=.22; *I*^2^=33%). Because of the very low quality of the evidence, the effects on quality of life and exercise adherence were uncertain. Apps did not influence the rate of adverse events.

**Conclusions:**

Apps providing personalized video exercises to support exercise performance significantly improved physical function and confidence in exercise performance. However, the level of evidence was low; more robust studies are needed to confirm these results.

**Trial Registration:**

PROSPERO CRD42022323670; https://www.crd.york.ac.uk/prospero/display_record.php?RecordID=323670

## Introduction

### Background

Disability is a major public health issue. Currently, more than 1 billion people (about 15% of the world’s population) live with short- and long-term disabling conditions [1]. In a lifetime, almost all individuals will experience a temporary or permanent disability. Health conditions leading to disability can affect a wide range of systems, such as the musculoskeletal, metabolic, cardiocerebral and vascular, nervous, or respiratory systems. In most cases, exercise programs are either the primary treatment or an essential adjunctive treatment to reduce short- and long-term disabling conditions [2,3]. In this document, short-term and long-term disabling conditions refer to people living with acute or chronic functional limitation, regardless of the location or type of impairment [4].

However, the effective implementation of exercise programs is limited by many factors. For health care professionals, the prescription of clear and detailed exercises can be difficult in the absence of adapted tools (eg, to transmit detailed exercise modalities). For individuals with short- and long-term disabling conditions, the first difficulty may be the availability of the health professional [5]. Then, when a program has been prescribed, other challenges may arise when performing exercises independently; the main challenge is to remember which exercises to perform and to stay motivated throughout the treatment process. As a consequence, the implementation of exercise programs is generally suboptimal [6].

In recent years, we have seen the emergence of mobile health technologies (mHealth), which may be very useful to facilitate the implementation of exercise programs [6]. Mobile health, or mHealth, is a subset of eHealth and is defined as “the use of mobile wireless technologies for health” [6]. mHealth offers easily accessible interventions that can reach large populations. In addition, interventions are low cost, easily adapted, reduce costs (such as travel), and save time [7-9]. The recent pandemic has highlighted the need and potential for telehealth solutions in situations in which travel is limited, and the health system is restricted [10]. These solutions can take the form of web-based platforms or apps offering physical exercise programs (generic or individualized to the person’s needs) prescribed by a health care professional or automatically provided by an algorithm embedded in the app [11]. One type of solution may be particularly useful in helping the therapist prescribe exercises and promote patient compliance: digital apps that allow us to design personalized exercise programs (individualized to the person’s needs), prescribed by a health professional (unlike programs proposed by algorithms, which cannot propose fine adjustments), and are accessible in the form of videos for a more faithful execution of the exercises. We will call such apps: apps providing personalized exercise videos [2,12,13]. To increase the use of these apps so that they may benefit as many people as possible, their effectiveness must be demonstrated [6,14].

To date, no systematic review has specifically evaluated the effectiveness of apps providing personalized video exercises. Existing reviews mix a wide variety of digital solutions, ranging from texting and phone calls to rehabilitation sessions via videoconference [7,11,13,15-17]. However, the heterogeneity of the studies included in those reviews prevented firm conclusions from being drawn on the effectiveness of digital solutions [18].

### Objectives

This systematic review aimed to synthesize the recent scientific literature on the impact of apps providing personalized video exercises to support rehabilitation for people with short- and long-term disabling conditions, on functional capacity, confidence in exercise performance, health care consumption, health-related quality of life, adherence, and adverse events.

## Methods

### Overview

This systematic literature review was conducted in accordance with the PRISMA (Preferred Reporting Items for Systematic Reviews and Meta-Analyses) guidelines [19] (Multimedia Appendix 1) and the Cochrane Handbook of Systematic Reviews [20]. The review protocol was registered with the International Prospective Register of Systematic Reviews (PROSPERO reference CRD42022323670) before commencement of the study. Deviations from the protocol are presented in Multimedia Appendix 2 [12,21,22].

### Eligibility Criteria

#### Population

The review covered people with any short- and long-term disabling conditions requiring an exercise program. To use an app independently, they had to be aged 11 years or older. Children aged 6-11 years required adult supervision according to EU regulations. The health conditions included were musculoskeletal system conditions, metabolic system conditions, cardio-cerebro vascular system conditions, nervous system conditions, respiratory system conditions, urinary system conditions, and cancers. Studies that required participants to be systematically accompanied by a caregiver, studies of healthy participants, or studies in which participants could not use the apps independently because of their pathology were excluded.

#### Intervention

This review included all apps providing personalized video exercises to support rehabilitation. The exercise program had to be prescribed by a health professional and tailored to the participant’s needs. The objective of the program had to be to increase physical function through addressing adherence to the exercise program (eg, programs aimed at educating participants without seeking to increase physical exercises). The app needed to be used under the indirect supervision of a health professional, with feedback possibilities. In addition, the app had to be a central element of the intervention being evaluated, not an additional component. Similarly, personalized video exercises were to be one of the main components of the app.

The following cases were not included in this review: studies evaluating only rehabilitation sessions via videoconferencing, exercise programs that were not prescribed and adapted by health care professionals (eg, program determined by an algorithm), and exercise programs available only on a website and not on an app.

#### Comparator

The comparator group included in this review was any type of control group (ie, waiting list, usual care or minimal interventions, or alternative treatment) as long as participants did not use apps providing personalized video exercises to support rehabilitation. The allocation of cointerventions between the intervention and comparison groups was allowed if the measured effect could be attributed to the exercise prescription app. Therefore, studies including other principal components in the intervention in addition to the app that were not proposed in the control group were excluded.

#### Outcomes

The primary outcome was functional capacity evaluated with tools including Oswerstry disability index, QuickDASH, or Modified Barthel Index. When more than one assessment of function was reported, self-reported questionnaires were preferred over performance tests because the questionnaire is usually more often used in the case of remote treatment, allowing remote assessment, which is not convenient for the performance test. Secondary outcomes included confidence in exercise performance (eg, self-efficacy), care consumption, health-related quality of life, adherence with the prescribed program, and adverse events, evaluated in both the intervention and comparator groups. We analyzed the results at the end of the intervention; we did not consider results collected during follow-up due to poor reporting in the preliminary research.

#### Study Design

This review included only randomized controlled trials. Studies for which the full text was not available or insufficient data were provided despite contact with the authors were excluded.

### Data Sources

We systematically searched the following databases: MEDLINE, CENTRAL, and Embase until March 1, 2022. We also searched clinical trial registries to identify ongoing studies, unpublished studies, and published studies not identified by the electronic search: ClinicalTrials.gov and WHO international clinical trials registry platform. We also searched the PROSPERO international prospective register for systematic reviews. Manufacturer’s websites of apps were searched (Physitrack, Kaia, Hinge, Swordhealth, and Caspar-health). One journal in the field (JMIR) was specifically searched. We manually searched the reference lists, studies that cited relevant studies as well as related studies in PubMed to identify any additional studies.

### Search Strategy

The search equations for the different databases are described in Multimedia Appendix 3. These search terms were extended with specific terminology and synonyms using Boolean operators and the respective Medical Subject Headings. No specific filters or limits were used, and the language was not restricted.

### Selection Process

The web-based data manager Rayyan.ai (Rayyan) was used for the selection process. After removal of duplicates, selection of relevant trials was performed by 2 independent authors (TD and PM) following the eligibility criteria defined above. The selection was made on the basis of title, summary, and then full text individually. A consensus was then sought on the papers finally selected. A third person was to be contacted in case of disagreement on the consensus but this was not necessary.

### Data Collection Process

Data were independently extracted by 2 authors (TD and PM) using a data extraction form previously tested with 3 trials [12,23,24]. When data were missing or unclear, we contacted the corresponding author for clarification. Information related to the intervention and the app was gathered from information available in the paper and the manufacturer’s website.

### Data Items

The data extracted included general characteristics (authors, year, study design, country of origin, and context of inclusion), the study population (pathology, number of participants, health conditions duration, body mass index, age, and sex), intervention details (app used and mode of delivery, duration, whether the device was currently on the market or not, components related to the digital therapeutic tool, and the exercise dosage), and general information about comparator group and outcome measures used in relation to our objectives (the measurement tool and the corresponding scale, the evaluation time at the end of the intervention and the mean, SD, and number of participants analyzed in each group at the end of the intervention). If there was an imbalance at baseline, we collected the difference between baseline and end of the intervention in each group. When several analyses were presented for the same outcome and assessment time point, we extracted the data from the intention-to-treat analysis and the analysis that used the most robust missing data imputation technique.

### Risk of Bias Assessment

The methodological quality of the included studies was assessed using the Cochrane Risk of Bias tool for quality assessment of randomized controlled trials (RoB 2) by 2 independent authors (TD and PM) [25]. Any discrepancies were resolved by discussion. To account for bias caused by deviations from the intended interventions, this review focused on the effect of assignment to the interventions at baseline (regardless of whether the interventions were received, or the level of participant adherence during follow-up, known as the “intention-to-treat effect”). The risk of bias was assessed according to the following domains: bias arising from the randomization process, bias due to deviations from intended interventions, bias due to missing data, bias in measurement of outcomes, and bias in selection of the reported result. The RoB 2 Excel tool was used to complete the risk of bias assessment. We judged each outcome as being at low risk, some concerns, or high risk according to the RoB 2 algorithm. The risk of bias assessment was incorporated in the Results section of the review, and it was also part of the Grading of Recommendations, Assessment, Development, and Evaluations (GRADE) assessment of the certainty of evidence (along with precision, directness, consistency, and publication bias).

### Effect Measures

For quantitative outcomes, the magnitude of effect was assessed with the mean difference (MD) when possible or with the SMD. Data from medians and IQRs were converted to means (SDs) [26]. For binary outcomes, the measure of effect was a risk ratio.

### Synthesis Methods

If the data were sufficiently homogeneous, the effects of the interventions were pooled using random effects models in RevMan (version 5.3; Cochrane Collaboration). To interpret the SMD, the following thresholds were used: no effect (<0.2), small effect (0.2-0.5), moderate effect (0.5-0.8), or large effect (>0.8) [27]. Heterogeneity was evaluated visually on the forest plots, using the heterogeneity test (*P*<.05 indicating significant heterogeneity) and *I*^2^ statistic that measures the proportion of variation (ie, inconsistency) between studies that is caused by heterogeneity rather than chance [28]. An *I*^2^ value of 0% to 40% might not be important; 30% to 60% may represent moderate heterogeneity; 50% to 90% may represent substantial heterogeneity; and 75% to 100% considerable heterogeneity [20]. Where there were insufficient data to pool studies, a narrative synthesis of the studies was conducted. As there were fewer than 10 trials per meta-analysis, we were unable to draw a funnel plot and perform an Egger test to assess small study effect [29].

### Grading the Level of Evidence

The quality of evidence for each outcome was assessed using GRADE to evaluate the following domains: study limitation (risk of bias), inconsistency, indirectness, imprecision, and publication bias [30].

## Results

### Study Selection and Characteristics

**Figure 1:**
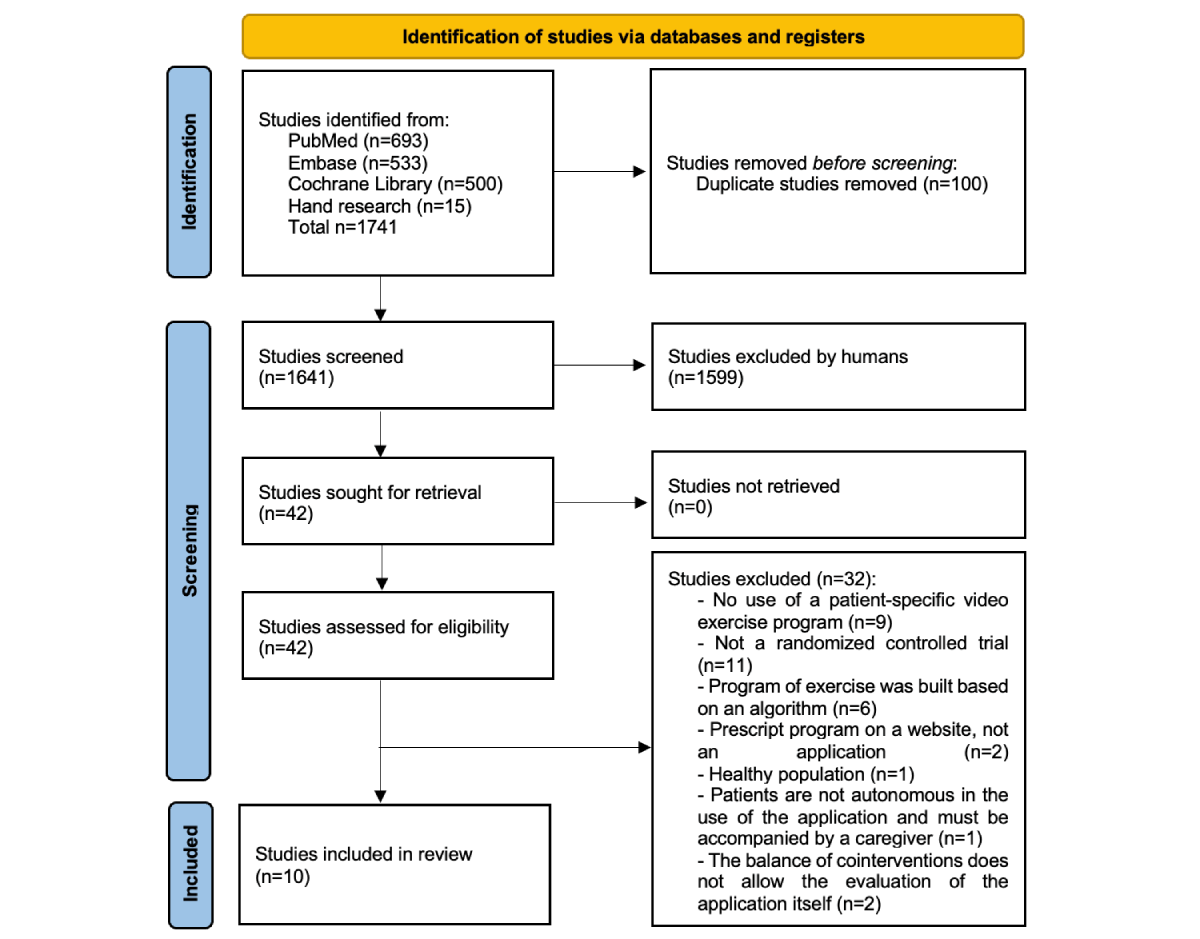
Flowchart of study selection and inclusion.

A total of 1641 titles and abstracts were screened after excluding duplicates, of which 1599 records did not meet the inclusion criteria (Figure 1). The full texts of 42 potential eligible records were read, and 10 papers published between 2017 and 2021 were included [12,21-24,31-35]. The list of papers excluded at the full-text selection stage with reasons for exclusion is presented in Multimedia Appendix 4. The 10 selected papers included a total of 1050 individuals (range across studies 20-305). A summary of the study characteristics is presented in Table 1. Eight studies involved adults, 1 involved children (aged 6 to 17 years) [32], and 1 involved people aged >60 years [23]. Overall, 7 studies included people with musculoskeletal disorders, and 3 included people with neurological disorders. Among these, 2 studies concerned employees and their dependents [22,24], and 3 involved rehabilitation after surgery [31,34,36].

**Table 1:** Participant characteristics of included studies.

Authors, country of origin	Condition	Context	Health conditions duration	Age (years), mean (SD)	Men (%)	BMI, mean (SD)	Participants included (IG^a^/CG^b^), n
Bennell et al [8], Australia and New Zealand	Adults with musculoskeletal condition	Physical therapist in private practice	<3 months (44%) >3 months (66%)	44.0 (15.0)	42	N/A^c^	305 (153/152)
Bui et al [31], Australia	Inpatients receiving routine orthopedic rehabilitation care	Private rehabilitation facility in the Sydney metropolitan	N/A	65.6 (16)	40	N/A	20 (10/10)
Correia et al [36], Portugal	Adults with shoulder rehabilitation after arthroscopic rotator cuff repair	Hospital da Prelada, Porto, Portugal	N/A	60.71 (6.9)	22	28.3 (4.9)	50 (27/23)
Ehling et al [21], Austria	Multiple sclerosis with moderate spasticity (≥4 on a normative rating scale)	Clinic for Neurological Rehabilitation Münster, Austria	Mean 14.4 (SD 3.4) years	48.5 (3.5)	55	N/A	20 (10/10)
Ellis et al [35], United States	Adults with mild to moderate Parkinson disease, not exercising over the past 3 months	Boston University Medical Center, Boston University, Center for Neurorehabilitation, and Fox Trial Finder	Mean 4.8 (SD 3.1) years	64.1 (9.5)	55	N/A	51 (26/25)
Hou et al [34], China	Adults who underwent lumbar spinal surgery	Three hospitals affiliated to the Sun Yat-sen University in China	N/A	50.0 (9.5)	53	N/A	168 (84/84)
Johnson et al [32], Australia	Children aged 6 to 17 years, with neurodevelopmental disabilities including cerebral palsy	Physiotherapy services registered with Australian Health Practitioner Regulation Agency	N/A	11.6 (3.3)	54	N/A	53 (26/27)
Li et al [37], Hong Kong	People 65 years or older with hip fracture, post–hip fracture surgery	Geriatric day hospital in a convalescent hospital in Hong Kong	<12 weeks	79.3 (9.2)	19	N/A	31 (15/16)
Mecklenburg et al [22], United States	Adults with chronic knee pain (>1 month in the last 12 months)	Employees and their dependents in over 12 office locations in the United States	>4 weeks	46.0 (12.0)	63	27.0 (5.0)	155 (101/54)
Shebib et al [24], United States	Adults with low back pain	Employees and their dependents at participating employers, across 12 locations in the United States	>6 weeks	43.0 (11.0)	59	26.0 (5.0)	177 (113/64)

^a^IG: intervention group.

^b^CG: control group.

^c^N/A: not available.

### Intervention and Comparator

A summary of the characteristics of interventions is presented in Table 2. The duration of intervention ranged from 3 to 48 weeks. All intervention groups received an exercise program with videos, tailored to each participant’s needs and available on an app. All trials except one [31] allowed communication between participants and therapists through the app. Exercise programs were designed by a physiotherapist in 8 studies [12,21,22,24,31,35,36,38], by a medical doctor in 1 study [34], and by an occupational therapist in 1 study [23]. Three studies provided real-time biofeedback through wearable motion sensors for the group using the app [22,24,36], 1 study provided feedback on steps per day, graphically over time for the intervention group and with simple information on the number of steps per day for the control group [35]. Seven studies used commercially available apps [12,22,24,35-38]. Six of the 10 included studies involved a comparison group receiving a similar exercise program as the intervention group but without the app (eg, on paper and with screenshots) [12,21,31,35,37,38]. The other 4 studies involved comparator groups that were significantly different from the intervention groups: more supervised sessions for 1 [36] and fewer components for 3 [22,24,34].

**Table 2:** Characteristics of interventions evaluated by included studies.

Authors	Intervention; program design; automatic feedback; mode of delivery and trackers; duration	Comparator	Supplementary information on exercise program
Bennell et al [12]	Usual PT^a^ care + home exercise program with app; PT; no; computer, tablet, or smartphone; 3 weeks	Usual PT care + home exercise program without app (printed exercise)	Includes educational material, self-reported PRO^b^, and activity planner or reminder.
Bui et al [19]	Usual care + home exercise program with app (range of motion, stretching, strengthening, and practice of everyday tasks); PT; no; Apple iPad Air 2; 4 weeks	Usual care + home exercise (paper or verbally)	Includes range of motion, stretching, strengthening, and practice of everyday task (eg, walking or standing up).
Correia et al [36]	Home exercise program with app (5 per week) + 13 home-based PT sessions (60 min); PT; real-time biofeedback through wearable motion sensors; tablet computer + 3 inertial motion trackers + activity tracker; 12 weeks	Home exercise program without app (>2 per week) + 36 home-based PT sessions (3 weeks, 30-60 min)	Five stages: (1) immediate postsurgery phase (weeks 0-2); (2) immobilization period (weeks 3-4); (3) passive mobilization (weeks 5-8); (4) active movement (weeks 9-10), and (5) strengthening (weeks 11-12). All at least 5 times per week for 15 to 30 min. Mobile app includes self-reported PRO and activity planner or reminder.
Ehling et al [21]	Home exercise program with app (6 per week, 2×15 min); PT; no; tablet; 12 weeks	Home exercise program without app (screenshots, 30 min daily)	Exercise program focused on movement, strengthening, and coordination of lower limbs and trunk, 2×15 min per day, 6 times per week. Mobile app includes activity planner or reminder.
Ellis et al [35]	Home exercise program with app + walking program with an advanced activity tracker; PT; steps per day through activity tracker (IG^c^/CG^d^); iPad; 48 weeks	Home exercise program without app + walking program with a simple pedometer	Exercise program includes strengthening and stretching. Mobile app includes self-reported PRO and activity planner or reminder.
Hou et al [34]	Usual care + home exercise program with app (20 min, twice a day); MD^e^; feedback about the rehabilitation; mobile app: 12 weeks	Usual care + advice to keep physically active and simple instructions to train the back muscles	Mobile app includes activity planner or reminder.
Johnson et al [32]	Home exercise program with app; PT; no; website or mobile app; 8 weeks	Home exercise program (handwritten, typed, or photo-program)	Mobile app includes self-reported PRO and activity planner or reminder.
Li et al [37]	Usual care (OT^f^ 110 min, 2 per week; PT; nursing care and consultations with a medical doctor in the day hospital) + unsupervised exercise program with app; OT; no; mobile app; 3 weeks	Usual care (same) + unsupervised exercise program through paper	Exercises aiming to improve trunk and lower limb strength, mobility, coordination, and balance. Functional exercises that were related to the daily living activities in the home environment. Includes educational material.
Mecklenburg et al [22]	Usual care + education papers (1 or 2 per week) + aerobic activities (30 min, 3 per week) + logging symptoms (2 per week) + cognitive behavioral therapy (1 per week) + weight loss program (if overweight, 1 per week) + home exercise program with app; PT designer but supervised by PC; real-time biofeedback through wearable motion sensors; tablet + 2 sensors to be used on the upper and lower leg; 12 weeks	Usual care + 3 education pieces regarding self-care for chronic knee pain	Exercise program includes standing quad stretch (pulling heel toward buttocks), seated quad stretch (pulling leg toward chest), half squats, forward lunges, leg raise (raising lower leg behind the body until parallel with floor while holding chair), seated leg raise (raising lower leg to horizontal while seated), and hamstring stretch (foot on raised object, reach to touch toes with straight leg). Includes educational material and activity planner or reminder.
Shebib et al [24]	Usual care + education papers (1 or 2 per week) + aerobic activities (30 min, 3 per week) + logging symptoms (2 per week) + cognitive behavioral therapy (1 per week) + home exercise program with app; PT designer but supervised by PC^g^; real-time biofeedback through wearable motion sensors; tablet + 2 sensors to be used on the upper and lower leg; 12 weeks	Usual care + 3 education pieces regarding self-care chronic pain	Exercise program includes standing quad stretch (pulling heel toward buttocks), seated quad stretch (pulling leg toward chest), half squats, forward lunges, leg raise (raising lower leg behind the body until parallel with floor while holding chair), seated leg raise (raising lower leg to horizontal while seated), and hamstring stretch (foot on raised object, reach to touch toes with straight leg). Includes educational material and activity planner or reminder.

^a^PT: physiotherapist.

^b^PRO: patient-reported outcome.

^c^IG: intervention group.

^d^CG: control group.

^e^MD: medical doctor.

^f^OT: occupational therapist.

^g^PC: personal coach.

### Risk of Bias

The results of the risk of bias assessment of the included studies are presented in Table 3. All included trials were rated as high risk of bias for at least 1 domain. The 2 domains with the highest proportion of high risk of bias were “bias due to deviations from intended interventions” and “bias in measurement of outcomes” because of the impossibility of blinding. The 2 domains with the highest proportion of low risk of bias were “bias arising from the randomization process” and “bias in selection of the reported result.”

**Table 3:** Risk of bias assessment of included studies using the Cochrane Risk of Bias tool 2 (N=10).

	Bias arising from the randomization process	Bias due to deviations from intended interventions	Bias due to missing data	Bias in measurement of outcomes	Bias in selection of the reported result
Bennell et al [12]	Low	Some concerns	Low	High	Low
Bui et al [31]	Low	High	Low	High	Low
Correia et al [36]	Low	High	Some concerns	High	Low
Ehling et al [21]	Some concerns	High	High	High	Some concerns
Ellis et al [35]	Some concerns	High	Some concerns	Low	Some concerns
Hou et al [34]	Low	High	High	High	Low
Johnson et al [32]	Low	High	Low	High	Low
Li et al [37]	Low	High	Low	High	Low
Mecklenburg et al [22]	Low	High	High	High	Low
Shebib et al [24]	Low	High	High	High	Low

### Overview of the Effects of Interventions

**Figure 2:**
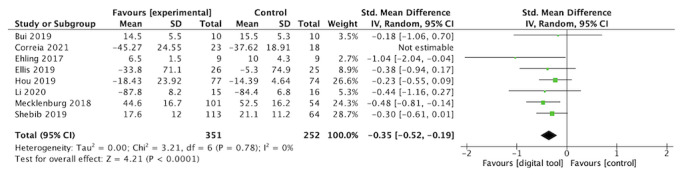
Forest plot of functional capacity [21, 22, 24, 34-38].

A summary of findings for the comparisons with GRADE ratings is presented in Table 4. Detailed meta-analytic forest plots are also presented in Figure 2 and Multimedia Appendix 5.

**Table 4:** Summary of findings with the level of evidence for each outcome.

Outcomes and time point measurement	Participants, n (studies)	Quality of the evidence^a^ (GRADE^b^)	Anticipated absolute effects^c^ (95% CI)	
			Risk with usual care or comparator group	Risk with digital apps providing personalized video-based exercise program
Physical function assessed with pooled outcome measures, follow-up: range 3 to 48 weeks	644 (8 RCTs^d^)	Low^e,f^	—^g^	SMD^h^ 0.35 (0.51 to 0.19)
Quality of life assessed with SMD, follow-up: range 12 to 48 weeks	237 (3 RCTs)	Very low^e,f,i,j^	—	SMD 0.42 (−0.09 to 0.93)
Adherence, follow-up: range 3 to 48 weeks	510 (6 RCTs)	Very low^e,f,i^	3 studies including 375 participants reported a statistical difference between intervention and control groups, while 3 other studies including 135 participants reported no significant difference	—
Confidence in ability to undertake exercise, scale from 0 to 10, follow-up: range 3 to 8 weeks	358 (2 RCTs)	Low^e,f^	The mean confidence in ability to undertake exercise was 0	MD^k^ 0.67 (0.37 to 0.96)
Health care consumption, follow-up: range 3 to 12 weeks	637 (3 RCTs)	Low^e,f^	The 3 studies involving 637 participants reported a statistical difference between intervention and control groups	—
Adverse events, follow-up: range 3 to 48 weeks	959 (7 RCTs)	Low^e,f^	56 per 1000	38 per 1000 (21 to 69)

^a^GRADE: Working group grades of evidence: high quality: further research is very unlikely to change our confidence in the estimate of effect; moderate quality: further research is likely to have an important impact on our confidence in the estimate of effect and may change the estimate; low quality: further research is very likely to have an important impact on our confidence in the estimate of effect and is likely to change the estimate; very low quality: we are very uncertain about the estimate.

^b^GRADE: Grading of Recommendations, Assessment, Development, and Evaluations.

^c^The risk in the intervention group (and its 95% CI) is based on the assumed risk in the comparison group and the relative effect of the intervention (and its 95% CI).

^d^RCT: randomized controlled trial.

^e^Most studies had a high risk of bias.

^f^Different outcomes and populations involved.

^g^Not available.

^h^SMD: standardized mean difference.

^i^Significant heterogeneity.

^j^Sample size <400 in standardized mean difference analysis.

^k^MD: mean difference.

### Effects of Interventions on Physical Function

Eight studies involving 644 participants reported the effect of an app providing personalized video exercises to support rehabilitation compared with the absence of apps on physical function at the postintervention time point (range 3 to 48 weeks, median 12 weeks; Figure 2). Details on how physical function was evaluated in included trials are reported in Multimedia Appendix 6. Overall, a significant small effect in favor of the intervention was observed (SMD 0.35, 95% CI 0.19-0.51); heterogeneity was low (*P*het=.86; *I*^2^=0%). Using the GRADE approach, the quality of evidence was rated as low because of the high risk of bias in most studies.

### Effects of Interventions on Confidence in Ability to Undertake Exercise

Two trials (358 participants) assessed confidence in the ability to undertake exercise on an 11-point numerical rating scale (0=strongly disagree and 10=strongly agree). Overall, a significant, small effect was found in favor of the intervention (MD 0.67, 95% CI 0.37-0.96); the heterogeneity was moderate (*P*het=.22; *I*^2^=33%). The quality of evidence was rated low because of the high risk of bias in most studies.

### Effects of Interventions on Health Care Consumption

Health care consumption was assessed in 3 studies (637 participants). All reported a statistical difference in favor of the intervention group. No meta-analysis was carried out owing to the high heterogeneity of the outcomes (Multimedia Appendix 7 [12,22,24]).

### Effects of Interventions on Health-Related Quality of Life

Overall, no significant difference was found between groups in the 3 studies assessing health-related quality of life (237 participants; SMD 0.42, 95% CI −0.09 to 0.93); heterogeneity was substantial (*P*het=.08; *I*^2^=60%). The quality of evidence was rated very low because of the high risk of bias in most studies, small sample size, and substantial heterogeneity.

### Effects of Interventions on Adherence

Among the 6 studies assessing adherence, 3 studies (n=375 participants) reported a statistical difference between intervention and control groups, while the 3 others (n=135 participants) reported no significant difference. No meta-analysis was performed because the adherence criterion was measured differently each time (Table 4).

### Effects of Interventions on Adverse Events

Adverse events were assessed in 7 studies (n=959 participants). Overall, no significant difference was found between groups (risk ratio 0.68, 95% CI 0.37-1.23); heterogeneity was low (*P*het=.38; *I*^2^=5%). The quality of evidence was rated as low because of the high risk of bias in most studies. Most studies did not explicitly mention the search for adverse events in the Methods section or in the protocol. No serious adverse events were reported in the intervention group of the 7 studies.

## Discussion

This review evaluated the effect of a specific digital strategy to promote adherence to exercise programs, thereby theoretically improving the clinical condition of patients: digital apps used by health professionals to deliver personalized video exercise programs. The results showed that the use of digital therapeutic tools to support exercise performance significantly improved physical function and confidence in exercise performance and may have reduced care consumption, with low-quality evidence. Owing to the very low quality of the evidence, the effects of digital tools in improving health-related quality of life and exercise adherence were uncertain. No differences were found between groups for adverse events.

The intervention evaluated involved indirect supervision by a therapist, with the individual performing the exercise program relatively autonomously. In this situation, the app is used as an extension of the rehabilitation session with the aim of maintaining the benefits of rehabilitation without additional therapist time. This kind of app has the potential to address the lack of support that many individuals in rehabilitation experience, especially those with chronic disorders, and to optimize face-to-face time for the most fragile individuals. Indeed, the use of a digital app providing videos of personalized exercises could provide more rehabilitation without significantly increasing the therapist's load. The results of our review in favor of a greater improvement in functional capacity should encourage the use of apps. In addition, greater patient empowerment could lead to greater availability of physicians and health professionals to treat more patients or spend more time with the less autonomous or more fragile patients [39]. By being more autonomous, the patient can increase the amount of exercise performed and educational content accessed without the direct intervention of a health professional. Finally, the use of an app to encourage self-rehabilitation can be a strategy to improve the consistency and standardization of rehabilitation among professionals and to disseminate good practice in rehabilitation. Indeed, the history of exercise programs and educational content prescribed to patients can be consulted and exchanged between therapists. Furthermore, as hands-on techniques are impossible by app, rehabilitation programs necessarily involve active exercises and education. Thus, the results of this systematic review suggest a revision of the traditional method of rehabilitation care.

To our knowledge, our review is the only one to provide a synthesis of a very homogeneous type of health app, allowing a more realistic conclusion on the clinical benefits.

In total, 10 papers were included, involving 1050 patients with musculoskeletal disorders (7 studies) and neurological disorders (3 studies). This number illustrates the growing interest in this particular type of solution.

Our results are coherent with the only review that specifically looked at digital therapeutic tools for implementing exercise programs [40]. That review focused on individuals with knee osteoarthritis and involved a large variety of apps, including digital apps providing personalized video-based exercise programs to support rehabilitation, as well as other types of apps [40]. Of the 11 studies included, 10 reported a statistically significant between-group improvement in pain.

The results of our review are also consistent with reviews that included all types of digital health tools (not specifically those focused on exercise). In most reviews, the authors did not distinguish between apps (using indirect therapist supervision and thus little therapist time) and platforms using teleconsultations (involving real-time therapist intervention similar to face-to-face settings) [7,11,15-17,41]. It is interesting to note that when comparing the 2 types of interventions, similar efficacy, safety, adherence, and user perceptions were found for remote compared to face-to-face care, for example, for people with rheumatic and musculoskeletal conditions [9]. However, the quality of the evidence of studies included in the review was low.

This review has strengths and weaknesses. First, one of the strengths is that it follows a rigorous methodology: registration with PROSPERO, use of the Cochrane methodology, and reporting according to PRISMA guidelines. Regarding weaknesses, the participants enrolled in the studies included were relatively homogeneous, consisting mainly of White, middle-aged people from higher income countries. Further studies are needed in other settings to extend the external validity of digital therapeutic tools to support exercise prescription [11].

Second, the quality of the studies included in this review was low, mostly due to deviations from the intended interventions and measurement of the outcome. The reason for this is that blinding in this kind of study is very challenging. Because of the risk of bias, we downgraded the level of evidence according to the GRADE tool. However, although the use of observational studies is currently encouraged to provide evidence of effectiveness in real-life situations, this review considered only the results of randomized controlled trials as these are currently the gold standard for the evaluation of effectiveness of digital therapeutic tools [42,43].

Third, this review included people with all types of disabling conditions to determine a common effect of app use, regardless of pathology. This choice is supported by the finding of many common components affecting adherence to exercise programs. For example, supervision by a health professional positively influences adherence in many conditions [44,45]. The same is true for the use of technology: adaptation to each individual, integration into daily life, communication, and feedback, which are characteristics of the type of app tested [46,47].

Fourth, this study analyzed outcomes at the end of the interventions only. However, for 1 study, the end of intervention results were given 1 year after the start, indicating the long-term effects that can be achieved [35]. Although the study yielded a small and statistically nonsignificant distinction between the groups, the findings imply the plausibility of a lasting impact. Finally, we encountered difficulties assessing the impact of interventions using apps on adherence. This is because each study proposed a particular type of measure to define adherence. Furthermore, 1 study did not report how adherence was evaluated [36], and 2 studies did not use the same method between the intervention and comparator groups [31,36]. It is interesting to note that no optimal measurement tool has been defined for the measurement of exercise adherence [48]. Owing to the high variability, we proposed a narrative synthesis rather than a meta-analysis. This additional limitation was considered in the GRADE analysis. It should be noted that for this study, the focus was on adherence to therapeutic exercises rather than engagement to the app itself.

The studies included in this review investigated the superiority of using a mobile app versus no app. Most comparator groups involved paper-based exercise prescriptions. However, 1 study compared the effect of the app with additional sessions supervised by a health professional. This type of study would benefit from seeking noninferiority rather than superiority because of the difference in the means deployed between the groups [36]. Nevertheless, we considered this trial to be a superiority study, even though the expected difference between the groups is necessarily more modest.

All the studies included in this review had in common the evaluation of the use of a mobile app to guide rehabilitation under asynchronous use of care, compared to no app. In some studies, the intervention group received a substantial number of cointerventions compared to the comparator group, which limits the interpretation of the results in favor of the effectiveness of the mobile app itself. Two studies were particularly affected by this cointervention imbalance [22,24]. However, we performed a sensitivity analysis to compare the effect of these 2 studies with that of the other studies. We found no difference in effect size compared to the other included studies on the primary outcome (data not shown).

From a clinical point of view, this review contributes to the awareness of the use of apps providing personalized video exercises by exploring their usefulness in supporting individuals in their rehabilitation outside the health care setting, allowing indirect supervision, and increasing rehabilitation time without increasing travel and the number of sessions. Our results suggest that rehabilitation can be extended beyond face-to-face sessions and the usual places of care provision thanks to these tools.

More studies are needed to confirm the effectiveness of apps providing personalized video exercises. In particular, this review highlighted the need for good quality randomized controlled trials assessing long-term benefits in diverse contexts and populations and evaluating exercise adherence and adverse events in a standardized manner. We encourage future reviews to focus on a homogeneous group of apps providing personalized video exercises to allow for greater consistency in the clinical conclusions drawn. Furthermore, the interventions evaluated in this review suggest that the effect of the app itself is limited without the intervention of a health professional providing direct or indirect supervision, answering questions outside the sessions, and adapting the exercises to the needs of each individual during the program. It seems necessary to define the components of an effective intervention to promote self-education, including the use of an app but also the involvement of the therapist.

To conclude, digital apps seem to support rehabilitation without increasing the rate of adverse effects. However, the level of confidence in the results remains low to very low; therefore, more robust studies are needed to confirm these results.

## Appendix

Multimedia Appendix 1 PRISMA (Preferred Reporting Items for Systematic Reviews and Meta-Analyses) 2020 statement checklist and related location in the paper.

Multimedia Appendix 2 Deviations from the protocol.

Multimedia Appendix 3 Electronic search developed for the 3 databases.

Multimedia Appendix 4 Description of nonincluded studies.

Multimedia Appendix 5 Detailed meta-analytic forest plots of secondary outcomes.

Multimedia Appendix 6 Assessment tools used in each study.

Multimedia Appendix 7 Additional information on health care consumption.

## Abbreviations

GRADE: Grading of Recommendations, Assessment, Development, and Evaluations
MD: mean difference
mHealth: mobile health
PRISMA: Preferred Reporting Items for Systematic Reviews and Meta-Analyses
PROSPERO: Prospective Register of Systematic Reviews
RoB 2: Risk of Bias
SMD: standardized mean difference
