# Correction: The Effectiveness of Digital Apps Providing Personalized Exercise Videos: Systematic Review With Meta-Analysis

**DOI:** 10.2196/52522

**Published:** 2023-09-21

**Authors:** Thomas Davergne, Philippe Meidinger, Agnès Dechartres, Laure Gossec

**Affiliations:** 1 Physical Medicine and Rehabilitation Department, Assistance Publique – Hôpitaux de Paris Lariboisière-Fernand-Widal, Université Paris Cité, Institut national de la santé et de la recherche médicale, Biologie de l'os et du cartilage Paris France; 2 Université Grenoble Alpes, Centre national de la recherche scientifique, VetAgro Sup, Grenoble Institut polytechnique de Grenoble Grenoble France; 3 Sorbonne Université, INSERM, Institut Pierre Louis d'Epidémiologie et de Santé Publique, AP-HP, Hôpital Pitié-Salpêtrière, Département de Santé Publique, 75013 Paris France; 4 Rheumatology Department, Pitié-Salpêtrière Hospital, Assistance Publique – Hôpitaux de Paris, Institut Pierre Louis d'Epidémiologie et de Santé Publique, Institut national de la santé et de la recherche médicale, Sorbonne Université Paris France

In “The Effectiveness of Digital Apps Providing Personalized Exercise Videos: Systematic Review With Meta-Analysis” (J Med Internet Res 2023;25:e45207) two errors were noted.

Affiliation of author Philippe Meidinger:

Recherche Translationnelle et Innovation en Médecine et Complexité, Unité Mixte de Recherche, Centre National de la Recherche Scientifique 5525, Théoriser et Modéliser pour Aménager Team, Université Grenoble Alpes, La Tronche, France

has been replaced by:

Université Grenoble Alpes, Centre national de la recherche scientifique, VetAgro Sup, Grenoble Institut polytechnique de Grenoble, Grenoble, France

And affiliation of author Agnès Dechartres:

Unité de Recherche Clinique Pitié Salpêtrière - Charles Foix, Centre de Pharmacoépidémiologie, Département de Santé Publique, Institut Pierre Louis d'Epidémiologie et de Santé Publique, Institut national de la santé et de la recherche médicale, Sorbonne Université, Paris, France

has been replaced by

Sorbonne Université, INSERM, Institut Pierre Louis d'Epidémiologie et de Santé Publique, AP-HP, Hôpital Pitié-Salpêtrière, Département de Santé Publique, 75013, Paris, France

The correction will appear in the online version of the paper on the JMIR Publications website on September 21, 2023, together with the publication of this correction notice. Because this was made after submission to PubMed, PubMed Central, and other full-text repositories, the corrected article has also been resubmitted to those repositories.

