# Supplementary material for: The Effectiveness of Digital Apps Providing Personalized Exercise Videos: Systematic Review With Meta-Analysis
Source: J Med Internet Res. 2023 Jul 13;25:e45207. doi: 10.2196/45207 (PMC10375281; doi:10.2196/45207)
Supplement: Multimedia Appendix 2 [file jmir_v25i1e45207_app2.docx]

| Search equations | The term "satisfaction" was removed from the equation. The term "functional capacity" was added. |
| --- | --- |
| Results on adherence | When several parameters of adherence were reported, we focused on the comparison between the groups that reflected the amount of exercise performed. |
| Results on quality of life | One study [29] reported SF36 as 2 sub-scores. The physical health sub-score was preferred for the meta-analysis over the mental health sub-score. |
| Result of health care consumption | For one study [31], the evaluation time chosen was the one closest to the end of the intervention. For one study [10], the number of co-interventions was preferred for the meta-analysis over the number of physiotherapy sessions to obtain a broader view |
