# Supplementary material for: The Effectiveness of Digital Apps Providing Personalized Exercise Videos: Systematic Review With Meta-Analysis
Source: J Med Internet Res. 2023 Jul 13;25:e45207. doi: 10.2196/45207 (PMC10375281; doi:10.2196/45207)
Supplement: Multimedia Appendix 3 [file jmir_v25i1e45207_app3.docx]

|  | For PubMed, until March 2022 |  |
| --- | --- | --- |
|  | Key words | Results |
| #1 | "online"[Title/Abstract] OR "mobile"[Title/Abstract] OR "app"[Title/Abstract] OR "web-based"[Title/Abstract] OR "digital"[Title/Abstract] OR "smartphone"[MeSH Terms] OR "mobile applications"[MeSH Terms] OR "Smartphones"[Title/Abstract] OR "smart phones"[Title/Abstract] OR "smart phone"[Title/Abstract] OR "phones smart"[Title/Abstract] OR "application mobile"[Title/Abstract] OR "applications mobile"[Title/Abstract] OR "mobile application"[Title/Abstract] OR "mobile apps"[Title/Abstract] OR "app mobile"[Title/Abstract] OR "apps mobile"[Title/Abstract] OR "mobile app"[Title/Abstract] OR "portable electronic apps"[Title/Abstract] OR ("App"[All Fields]) AND ("portable electronic"[Title/Abstract])) OR (("electronical"[All Fields] OR "electronically"[All Fields] OR "electronics"[MeSH Terms] OR "electronics"[All Fields] OR "Electronic"[All Fields]) AND "app portable"[Title/Abstract]) OR (("portability"[All Fields] OR "Portable"[All Fields] OR "portables"[All Fields]) AND "electronic app"[Title/Abstract]) OR "portable electronic applications"[Title/Abstract] OR (("applicabilities"[All Fields] OR "applicability"[All Fields] OR "Application"[All Fields] OR "Applications"[All Fields] OR "applicative"[All Fields]) AND "portable electronic"[Title/Abstract]) OR "electronic application portable"[Title/Abstract] OR "portable electronic application"[Title/Abstract] OR (("portability"[All Fields] OR "Portable"[All Fields] OR "portables"[All Fields]) AND "software apps"[Title/Abstract]) OR (("australas plant pathol"[Journal] OR "App"[All Fields]) AND "portable software"[Title/Abstract]) OR (("portability"[All Fields] OR "Portable"[All Fields] OR "portables"[All Fields]) AND "software app"[Title/Abstract]) OR (("Software"[MeSH Terms] OR "Software"[All Fields] OR "software s"[All Fields] OR "softwares"[All Fields]) AND "app portable"[Title/Abstract]) OR (("portability"[All Fields] OR "Portable"[All Fields] OR "portables"[All Fields]) AND "software applications"[Title/Abstract]) OR "application portable software"[Title/Abstract] OR "portable software application"[Title/Abstract] OR (("Software"[MeSH Terms] OR "Software"[All Fields] OR "software s"[All Fields] OR "softwares"[All Fields]) AND "application portable"[Title/Abstract]) OR "smartphone apps"[Title/Abstract] OR "app smartphone"[Title/Abstract] OR "apps smartphone"[Title/Abstract] OR "smartphone app"[Title/Abstract] | 522,623 |
| #2 | "Training"[Title/Abstract] OR "Exercise"[MeSH Terms] OR "rehabilitation"[MeSH Terms] OR ("Exercises"[Title/Abstract] OR "physical activity"[Title/Abstract] OR "activities physical"[Title/Abstract] OR "activity physical"[Title/Abstract] OR "physical activities"[Title/Abstract] OR "exercise physical"[Title/Abstract] OR "exercises physical"[Title/Abstract] OR "physical exercise"[Title/Abstract] OR "physical exercises"[Title/Abstract] OR "acute exercise"[Title/Abstract] OR "acute exercises"[Title/Abstract] OR "exercise acute"[Title/Abstract] OR "exercises acute"[Title/Abstract] OR "exercise isometric"[Title/Abstract] OR "exercises isometric"[Title/Abstract] OR "isometric exercises"[Title/Abstract] OR "isometric exercise"[Title/Abstract] OR "exercise aerobic"[Title/Abstract] OR "aerobic exercise"[Title/Abstract] OR "aerobic exercises"[Title/Abstract] OR "exercises aerobic"[Title/Abstract] OR "exercise training"[Title/Abstract] OR "exercise trainings"[Title/Abstract] OR "training exercise"[Title/Abstract] OR (("education"[MeSH Subheading] OR "education"[All Fields] OR "Training"[All Fields] OR "education"[MeSH Terms] OR "train"[All Fields] OR "train s"[All Fields] OR "trained"[All Fields] OR "training s"[All Fields] OR "Trainings"[All Fields] OR "trains"[All Fields]) AND "Exercise"[Title/Abstract])) | 961,272 |
| #3 | “Supervision”[All Fields] OR “prescription”[All Fields] OR “personali*”[All Fields] OR “customi*”[All Fields] OR “individually tailored”[All Fields] | 415,808 |
| #4 | "Adherence"[Title/Abstract] OR "quality of life"[Title/Abstract] OR "risk assessment"[Title/Abstract] OR "function"[Title/Abstract] OR "organization of care"[Title/Abstract] | 2,743,289 |
|  | #1 AND #2 AND #3 AND #4 | 529 |

|  | For Embase, until March 2022 |  |
| --- | --- | --- |
|  | Key words | Results |
| #1 | online OR mobile OR app OR (smartphone AND app) OR ‘web based’ OR digital OR 'smartphone'/exp OR 'smartphone' OR ((mobile or cell$ or cellular or smart) adj ((phone$1 adj2 app$1) or application$1)).ti,ab. oR smartphone$.ti,ab | 1,041,724 |
| #2 | exercise AND program* OR training OR rehabilitation OR 'exercise'/exp | 1,554,890 |
| #3 | supervision OR prescription OR personali* OR customi* OR (individually AND tailored) | 737,637 |
| #4 | adherence OR (quality AND of AND life) OR (risk AND assessment) OR function OR (organisation AND of AND care) | 5,966,569 |
|  | #1 AND #2 AND #3 AND #4 | 1563 |

|  | For the Cochrane library, until March 2022 | Results |
| --- | --- | --- |
| #1 | online OR mobile OR app OR smartphone app OR web-based OR digital OR smartphone* OR mobile* or smart near/2 phone* | 50610 |
| #3 | ((Exercise program*) OR (training) OR (rehabilitation)):ti,ab,kw | 156176 |
| #4 | (Supervision) OR (prescription) OR (personali*) OR (customi*) OR (individually tailored) | 45607 |
|  | #1 AND #2 AND #3 | 1383 |
