# Supplementary material for: The Effectiveness of Digital Apps Providing Personalized Exercise Videos: Systematic Review With Meta-Analysis
Source: J Med Internet Res. 2023 Jul 13;25:e45207. doi: 10.2196/45207 (PMC10375281; doi:10.2196/45207)
Supplement: Multimedia Appendix 4 [file jmir_v25i1e45207_app4.docx]

| **First author (year of publication)** | **Study design** | **Population** | **Comment** |
| --- | --- | --- | --- |
| Almhdawi (2020) [1] | Pilot randomized controlled trial | Office workers with low back pain | No use of a patient-specific video exercise program |
| Bossen (2016) [2] | Single-arm pilot study | Knee and Hip Osteoarthritis | Not a randomized controlled trial |
| Chhabra (2018) [3] | Randomized controlled trial | Chronic low back pain | No use of a patient-specific video exercise program |
| Choi (2018) [4] | Randomized controlled trial | Frozen shoulder | No use of a patient-specific video exercise program |
| Chung (2020) [5] | Randomized controlled trial | Stroke | Prescribed program on a website, not an application |
| Conijn (2020) [6] | Quasi-experimental pilot study | Adult patients undergoing elective organ transplantation or vascular surgery | Not a randomized controlled trial |
| Correia (2018) [7] | Non randomized parallel-group trial | Total knee arthroplasty | Not a randomized controlled trial |
| Correia (2019) [8] | Non randomized parallel-group trial | Total knee arthroplasty | Not a randomized controlled trial |
| Correia (2019) [9] | Non randomized parallel-group trial | Total hip arthroplasty | Not a randomized controlled trial |
| Cramer (2019) [10] | Randomized controlled trial | Stroke | No use of a patient-specific video exercise program |
| Daily (2021) [11] | Single-arm pilot study | Musculoskeletal Health in Community-Dwelling Older Adults | Healthy population |
| Emmerson (2017) [12] | Randomized controlled trial | Stroke | No use of a patient-specific video exercise program |
| Hasenöhrl (2020) [13] | Single-arm pilot study | Low back pain | Not a randomized controlled trial |
| Huber (2017) [14] | Retrospective longitudinal cohort study | Low back pain | Not a randomized controlled trial |
| Kim (2021) [15] | Single-arm pilot study | Parkinson Disease | Not a randomized controlled trial |
| Kloek (2018) [16] | Randomized controlled trial | Knee osteoarthritis | No use of a patient-specific video exercise program |
| Lambert (2017) [17] | Randomized controlled trial | Musculoskeletal conditions | No use of a patient-specific video exercise program |
| Landers (2020) [18] | Single-arm pilot study | Parkinson Disease | Program of exercise was built based on an algorithm |
| Lee (2016) [19] | Single-group, repeated-measures design | Office Workers With Neck Pain | Program of exercise was built based on an algorithm |
| Lo (2018) [20] | Cross-sectional questionnaire study | Chronic Neck and Back Pain | Program of exercise was built based on an algorithm |
| Paul (2014) [21] | Randomized controlled trial | People moderately affected with Multiple Sclerosis | Prescribed program on a website, not an application |
| Pinto (2021) [22] | Single arm cohort study | Cardiovascular disease | No use of a patient-specific video exercise program |
| Priebe (2020) [23] | Cluster randomized controlled trial | Non-specific back pain | The balance of co-interventions did not allow the evaluation of the application itself |
| Raiszadeh (2021) [24] | Non randomized parallel-group trial | Low back pain | Not a randomized controlled trial |
| Rassouli (2018) [25] | Observational pilot study | COPD | Not a randomized controlled trial |
| Sandal (2020) [26] | Single arm cohort study | Low back pain | Program of exercise was built by an algorithm |
| Smittenaar (2017) [27] | Single arm cohort study | Chronic knee pain | Not a randomized controlled trial |
| Spielmanns (2022) [28] | Randomized controlled trial | COPD | Program of exercise was built by an algorithm |
| Tabak (2014) [29] | Randomized controlled trial | Patients with COPD exacerbations | The balance of co-interventions did not allow the evaluation of the application itself |
| Toelle (2019) [30] | Randomized controlled trial | Non-specific low back pain | Program of exercise was built by an algorithm |
| Vloothuis (2019) [31] | Randomized controlled trial | Adults with stroke, and a caregiver who was willing and able to do exercises together with the person | Participants were not autonomous in the use of the application and had to be accompanied by a caregiver |
| Yang (2019) [32] | Randomized controlled trial | Chronic low back pain | No use of a patient-specific video exercise program |

References:

1. Spielmanns M, Gloeckl R, Jarosch I, Leitl D, Schneeberger T, Boeselt T, Huber S, Kaur-Bollinger P, Ulm B, Mueller C, Bjoerklund J, Spielmanns S, Windisch W, Pekacka-Egli A-M, Koczulla AR. Using a smartphone application maintains physical activity following pulmonary rehabilitation in patients with COPD: a randomised controlled trial. Thorax BMJ Publishing Group Ltd; 2022 Apr 21; PMID:35450945

2. Pinto R, Pires ML, Borges M, Pinto ML, Sousa Guerreiro C, Miguel S, Santos O, Ricardo I, Cunha N, Alves da Silva P, Correia AL, Fiúza S, Caldeira E, Salazar F, Rodrigues C, Cordeiro Ferreira M, Afonso G, Araújo G, Martins J, Ramalhinho M, Sousa P, Pires S, Jordão A, Pinto FJ, Abreu A. Digital home-based multidisciplinary cardiac rehabilitation: How to counteract physical inactivity during the COVID-19 pandemic. Rev Port Cardiol 2021 Nov 20; PMID:34840415

3. Kim A, Yun SJ, Sung K-S, Kim Y, Jo JY, Cho H, Park K, Oh B-M, Seo HG. Exercise Management Using a Mobile App in Patients With Parkinsonism: Prospective, Open-Label, Single-Arm Pilot Study. JMIR mHealth and uHealth 2021 Aug 31;9(8):e27662. doi: 10.2196/27662

4. Raiszadeh K, Tapicer J, Taitano L, Wu J, Shahidi B. In-Clinic Versus Web-Based Multidisciplinary Exercise-Based Rehabilitation for Treatment of Low Back Pain: Prospective Clinical Trial in an Integrated Practice Unit Model. Journal of Medical Internet Research 2021 Mar 18;23(3):e22548. doi: 10.2196/22548

5. Daly RM, Gianoudis J, Hall T, Mundell NL, Maddison R. Feasibility, Usability, and Enjoyment of a Home-Based Exercise Program Delivered via an Exercise App for Musculoskeletal Health in Community-Dwelling Older Adults: Short-term Prospective Pilot Study. JMIR mHealth and uHealth 2021 Jan 13;9(1):e21094. doi: 10.2196/21094

6. Landers MR, Ellis TD. A Mobile App Specifically Designed to Facilitate Exercise in Parkinson Disease: Single-Cohort Pilot Study on Feasibility, Safety, and Signal of Efficacy. JMIR mHealth and uHealth 2020 Oct 5;8(10):e18985. doi: 10.2196/18985

7. Almhdawi KA, Obeidat DS, Kanaan SF, Oteir AO, Mansour ZM, Alrabbaei H. Efficacy of an innovative smartphone application for office workers with chronic non-specific low back pain: a pilot randomized controlled trial. Clin Rehabil 2020 Oct;34(10):1282–1291. PMID:32602362

8. Priebe JA, Haas KK, Moreno Sanchez LF, Schoefmann K, Utpadel-Fischler DA, Stockert P, Thoma R, Schiessl C, Kerkemeyer L, Amelung V, Jedamzik S, Reichmann J, Marschall U, Toelle TR. Digital Treatment of Back Pain versus Standard of Care: The Cluster-Randomized Controlled Trial, Rise-uP. J Pain Res 2020 Jul 17;13:1823–1838. PMID:32765057

9. Conijn D, van Bodegom-Vos L, Volker W, Mertens B, Vermeulen H, Huurman V, van Schaik J, Vliet Vlieland T, Meesters J. A multicomponent intervention to decrease sedentary time during hospitalization: a quasi-experimental pilot study. Clin Rehabil SAGE Publications Ltd STM; 2020 Jul 1;34(7):901–915. doi: 10.1177/0269215520920662

10. Chung BPH, Chiang WKH, Lau H, Lau TFO, Lai CWK, Sit CSY, Chan KY, Yeung CY, Lo TM, Hui E, Lee JSW. Pilot study on comparisons between the effectiveness of mobile video-guided and paper-based home exercise programs on improving exercise adherence, self-efficacy for exercise and functional outcomes of patients with stroke with 3-month follow-up: A single-blind randomized controlled trial. Hong Kong Physiother J 2020 Jun;40(1):63–73. PMID:32489241

11. Sandal LF, Øverås CK, Nordstoga AL, Wood K, Bach K, Hartvigsen J, Søgaard K, Mork PJ. A digital decision support system (selfBACK) for improved self-management of low back pain: a pilot study with 6-week follow-up. Pilot and Feasibility Studies 2020 May 23;6(1):72. doi: 10.1186/s40814-020-00604-2

12. Hasenöhrl T, Windschnurer T, Dorotka R, Ambrozy C, Crevenna R. Prescription of individual therapeutic exercises via smartphone app for patients suffering from non-specific back pain. Wien Klin Wochenschr 2020;132(5):115–123. PMID:32060724

13. Cramer SC, Dodakian L, Le V, See J, Augsburger R, McKenzie A, Zhou RJ, Chiu NL, Heckhausen J, Cassidy JM, Scacchi W, Smith MT, Barrett AM, Knutson J, Edwards D, Putrino D, Agrawal K, Ngo K, Roth EJ, Tirschwell DL, Woodbury ML, Zafonte R, Zhao W, Spilker J, Wolf SL, Broderick JP, Janis S, National Institutes of Health StrokeNet Telerehab Investigators. Efficacy of Home-Based Telerehabilitation vs In-Clinic Therapy for Adults After Stroke: A Randomized Clinical Trial. JAMA Neurol 2019 Sep 1;76(9):1079–1087. PMID:31233135

14. Correia FD, Nogueira A, Magalhães I, Guimarães J, Moreira M, Barradas I, Molinos M, Teixeira L, Pires J, Seabra R, Lains J, Bento V. Digital Versus Conventional Rehabilitation After Total Hip Arthroplasty: A Single-Center, Parallel-Group Pilot Study. JMIR Rehabilitation and Assistive Technologies 2019 Jun 21;6(1):e14523. doi: 10.2196/14523

15. Toelle TR, Utpadel-Fischler DA, Haas K-K, Priebe JA. App-based multidisciplinary back pain treatment versus combined physiotherapy plus online education: a randomized controlled trial. npj Digit Med 2019 May 3;2(1):1–9. doi: 10.1038/s41746-019-0109-x

16. Vloothuis JDM, Mulder M, Nijland RHM, Goedhart QS, Konijnenbelt M, Mulder H, Hertogh CMPM, Tulder M van, Wegen EEH van, Kwakkel G. Caregiver-mediated exercises with e-health support for early supported discharge after stroke (CARE4STROKE): A randomized controlled trial. PLOS ONE Public Library of Science; 2019 Apr 8;14(4):e0214241. doi: 10.1371/journal.pone.0214241

17. Choi Y, Nam J, Yang D, Jung W, Lee H-R, Kim SH. Effect of smartphone application-supported self-rehabilitation for frozen shoulder: a prospective randomized control study. Clin Rehabil SAGE Publications Ltd STM; 2019 Apr 1;33(4):653–660. doi: 10.1177/0269215518818866

18. Correia FD, Nogueira A, Magalhães I, Guimarães J, Moreira M, Barradas I, Molinos M, Teixeira L, Tulha J, Seabra R, Lains J, Bento V. Medium-Term Outcomes of Digital Versus Conventional Home-Based Rehabilitation After Total Knee Arthroplasty: Prospective, Parallel-Group Feasibility Study. JMIR Rehabilitation and Assistive Technologies 2019 Feb 28;6(1):e13111. doi: 10.2196/13111

19. Yang J, Wei Q, Ge Y, Meng L, Zhao M. Smartphone-Based Remote Self-Management of Chronic Low Back Pain: A Preliminary Study. Journal of Healthcare Engineering Hindawi; 2019 Feb 6;2019:e4632946. doi: 10.1155/2019/4632946

20. Lo WLA, Lei D, Li L, Huang DF, Tong K-F. The Perceived Benefits of an Artificial Intelligence–Embedded Mobile App Implementing Evidence-Based Guidelines for the Self-Management of Chronic Neck and Back Pain: Observational Study. JMIR mHealth and uHealth 2018 Nov 26;6(11):e8127. doi: 10.2196/mhealth.8127

21. Chhabra HS, Sharma S, Verma S. Smartphone app in self-management of chronic low back pain: a randomized controlled trial. Eur Spine J 2018 Nov;27(11):2862–2874. PMID:30324496

22. Correia FD, Nogueira A, Magalhães I, Guimarães J, Moreira M, Barradas I, Teixeira L, Tulha J, Seabra R, Lains J, Bento V. Home-based Rehabilitation With A Novel Digital Biofeedback System versus Conventional In-person Rehabilitation after Total Knee Replacement: a feasibility study. Sci Rep 2018 Jul 26;8(1):11299. doi: 10.1038/s41598-018-29668-0

23. Kloek CJJ, Bossen D, Spreeuwenberg PM, Dekker J, de Bakker DH, Veenhof C. Effectiveness of a Blended Physical Therapist Intervention in People With Hip Osteoarthritis, Knee Osteoarthritis, or Both: A Cluster-Randomized Controlled Trial. Physical Therapy 2018 Jul 1;98(7):560–570. doi: 10.1093/ptj/pzy045

24. Rassouli F, Boutellier D, Duss J, Huber S, Brutsche MH. Digitalizing multidisciplinary pulmonary rehabilitation in COPD with a smartphone application: an international observational pilot study. Int J Chron Obstruct Pulmon Dis 2018;13:3831–3836. PMID:30538444

25. Emmerson KB, Harding KE, Lockwood KJ, Taylor NF. Home exercise programs supported by video and automated reminders for patients with stroke: A qualitative analysis. Australian Occupational Therapy Journal 2018;65(3):187–197. doi: 10.1111/1440-1630.12461

26. Huber S, Priebe JA, Baumann K-M, Plidschun A, Schiessl C, Tölle TR. Treatment of Low Back Pain with a Digital Multidisciplinary Pain Treatment App: Short-Term Results. JMIR Rehabilitation and Assistive Technologies 2017 Dec 4;4(2):e9032. doi: 10.2196/rehab.9032

27. Lambert TE, Harvey LA, Avdalis C, Chen LW, Jeyalingam S, Pratt CA, Tatum HJ, Bowden JL, Lucas BR. An app with remote support achieves better adherence to home exercise programs than paper handouts in people with musculoskeletal conditions: a randomised trial. J Physiother 2017 Jul;63(3):161–167. PMID:28662834

28. Smittenaar P, Erhart-Hledik JC, Kinsella R, Hunter S, Mecklenburg G, Perez D. Translating Comprehensive Conservative Care for Chronic Knee Pain Into a Digital Care Pathway: 12-Week and 6-Month Outcomes for the Hinge Health Program. JMIR Rehabilitation and Assistive Technologies 2017 Apr 5;4(1):e7258. doi: 10.2196/rehab.7258

29. Lee M, Lee SH, Kim T, Yoo H-J, Kim SH, Suh D-W, Son J, Yoon B. Feasibility of a Smartphone-Based Exercise Program for Office Workers With Neck Pain: An Individualized Approach Using a Self-Classification Algorithm. Arch Phys Med Rehabil 2017 Jan;98(1):80–87. PMID:27693421

30. Bossen D, Kloek C, Snippe HW, Dekker J, de Bakker D, Veenhof C. A Blended Intervention for Patients With Knee and Hip Osteoarthritis in the Physical Therapy Practice: Development and a Pilot Study. JMIR Res Protoc 2016 Feb 24;5(1):e32. PMID:26912378

31. Tabak M, Brusse-Keizer M, van der Valk P, Hermens H, Vollenbroek-Hutten M. A telehealth program for self-management of COPD exacerbations and promotion of an active lifestyle: a pilot randomized controlled trial. Int J Chron Obstruct Pulmon Dis 2014 Sep 9;9:935–944. PMID:25246781

32. Paul L, Coulter EH, Miller L, McFadyen A, Dorfman J, Mattison PGG. Web-based physiotherapy for people moderately affected with Multiple Sclerosis; quantitative and qualitative data from a randomized, controlled pilot study. Clin Rehabil 2014 Sep;28(9):924–935. PMID:24691218
