# Supplementary material for: The Effectiveness of Digital Apps Providing Personalized Exercise Videos: Systematic Review With Meta-Analysis
Source: J Med Internet Res. 2023 Jul 13;25:e45207. doi: 10.2196/45207 (PMC10375281; doi:10.2196/45207)

1. Health-related quality of life, follow-up: range 12 weeks to 48 weeks


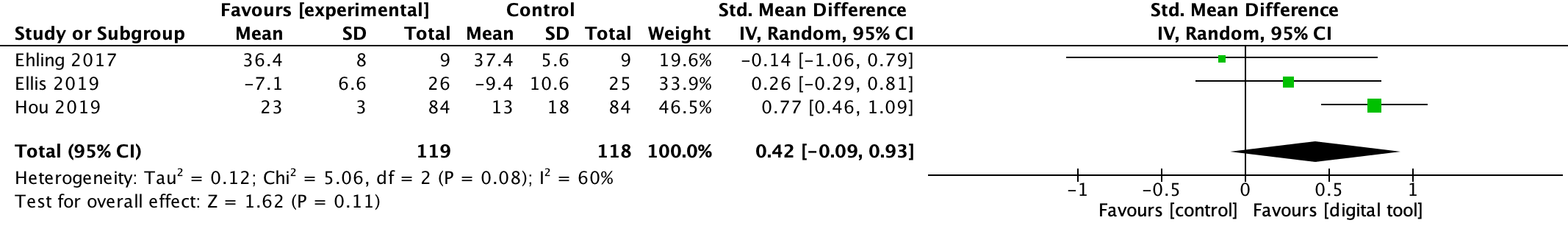


1. Confidence in ability to undertake exercise, scale from: 0 to 10, follow-up: range 3 weeks to 8 weeks


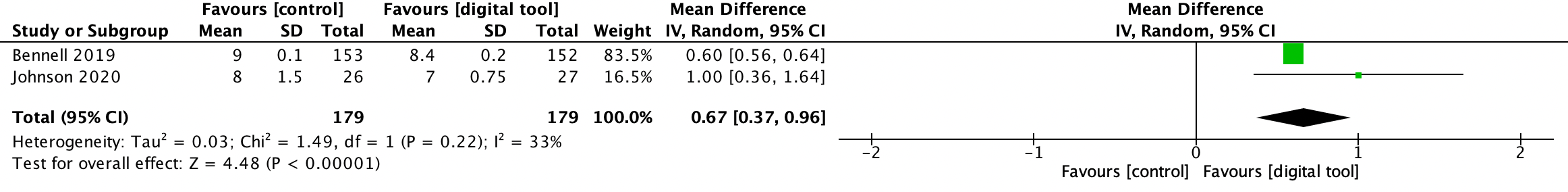


1. Adverse events, follow-up: range 3 weeks to 48 weeks


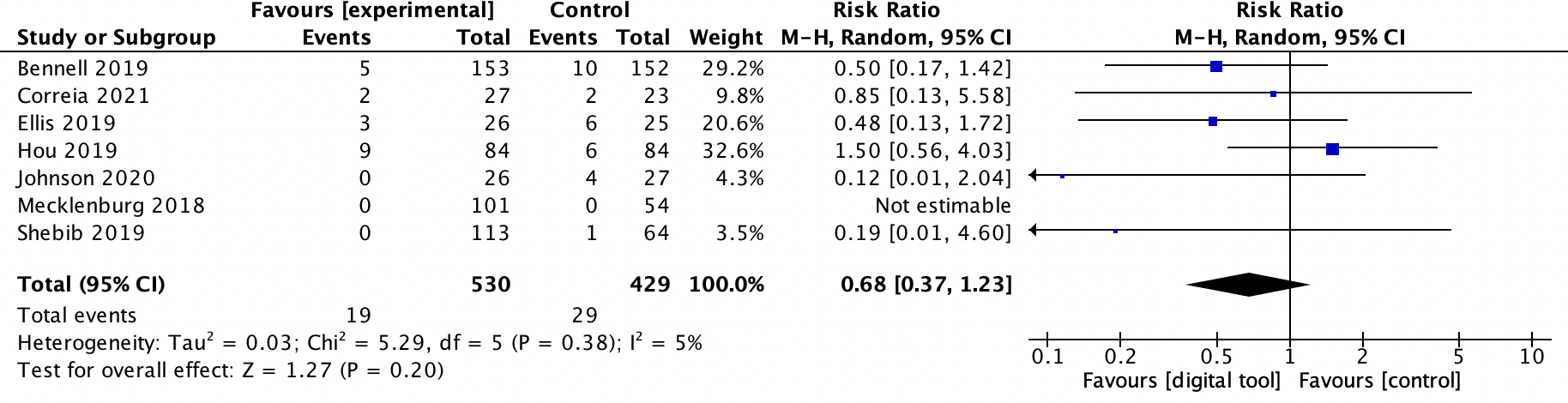

Supplement: Multimedia Appendix 5 [file jmir_v25i1e45207_app5.docx]
