# Supplementary material for: The Effectiveness of Digital Apps Providing Personalized Exercise Videos: Systematic Review With Meta-Analysis
Source: J Med Internet Res. 2023 Jul 13;25:e45207. doi: 10.2196/45207 (PMC10375281; doi:10.2196/45207)
Supplement: Multimedia Appendix 6 [file jmir_v25i1e45207_app6.docx]

| **Physical function** | |
| --- | --- |
| Bui 2019 | Functional Independence Measure |
| Correia 2021 | QuickDASH |
| Ehling 2017 | Timed 25-foot walk |
| Ellis | 6-Minute Walk Test |
| Ellis 2019 | 6-Minute Walk Test |
| Hou 2019 | Oswestry Disability IndexDI |
| Li 2020 | Modified Barthel Index |
| Mecklenburg 2018 | KOOS Physical Function Shortform |
| Shebib 2019 | Oswestry Disability IndexDI |
| **Confidence in exercise performance** | |
| Bennell 2019 | Confidence in ability to undertake exercise. 11-point Numerical Rating Scale |
| Johnson 2020 | Confidence in ability to undertake exercise. 11-point Numerical Rating Scale |
| **Use of care** | |
| Bennell 2019 | Number of participants receiving co-interventions |
| Mecklenburg 2018 | Knee surgery intent in the next year |
| Shebib 2019 | Back surgery intent |
| **Health-related quality of life** | |
| Ehling 2017 | SF36 physical health |
| Ellis 2019 | Parkinson Disease Questionnaire (PDQ-39) index |
| Hou 2019 | EQ5D |
| **Adherence** | |
| Bennell 2019 | Self-reported overall adherence (11-point Numerical Rating Scale) |
| Bui 2019 | Amount of time spent in exercise (minute) collected through app for intervention group and through diaries for control group. |
| Correia 2021 | Total treatment time (hours) |
| Johnson 2022 | Proportion of exercises attempted of total exercises prescribed (self-reported on exercise logbook) |
| Li 2020 | Completion of 90% of the home program |
| Ellis 2019 | Average number of days per week spent on the exercise program collected by the app for intervention group and on a calendar for control group |
| **Adverse events** | |
| Bennell 2019 | Any health problem that participants believed was caused by the exercise that required them to seek treatment/medication and/or interfered with function for two or more days |
| Correira 2021 | Adverse events registered during the rehabilitation programs in the patient’s files |
| Ellis 2019 | Safety was assessed by having a blinded research assistant call participants monthly to monitor adverse events and to ask a standard set of questions about recent health events and their relationship to the intervention. |
| Hou 2019 | Self-reported |
| Johnson 2020 | Self-reported |
| Mecklenburg 2018 | Self-reported |
| Shebib 2019 | Self-reported |
