# Supplementary material for: The Effectiveness of Digital Apps Providing Personalized Exercise Videos: Systematic Review With Meta-Analysis
Source: J Med Internet Res. 2023 Jul 13;25:e45207. doi: 10.2196/45207 (PMC10375281; doi:10.2196/45207)
Supplement: Multimedia Appendix 7 [file jmir_v25i1e45207_app7.docx]

Multimedia Appendix 7

Health care consumption was assessed in one study through the number of patients receiving co-interventions (13 out of 153 in intervention versus 31 out of 152 in the control group), the difference was statistically significant (*P*=.003) [10]. Another study assessed health consumption through the intention to undergo knee surgery in the next year on a 0–100-point scale. A significant difference was found between groups (intervention group: mean 14.7 [SD 25], control group mean 23.9 [SD 29.1], *P*=.01) [31]. A third study assessed health consumption through the intention to undergo back surgery on a 0–10-point scale, 10="definitely going to get surgery”). A significant difference was found between groups (intervention group: mean 0.62 [SD 1.35], control group: mean 1.53 [SD 2.67], *P*=.01) [32].
